# Influence of wind and light on the floating and sinking process of *Microcystis*

**DOI:** 10.1038/s41598-022-08977-5

**Published:** 2022-04-05

**Authors:** Zongpu Xue, Wei Zhu, Yuyang Zhu, Xihui Fan, Huaimin Chen, Ganyu Feng

**Affiliations:** 1 College of Hydrology and Water Resources, Hohai University, Nanjing, 210098 People’s Republic of China; 2 College of Environment, Hohai University, Nanjing, 210098 People’s Republic of China; 3 College of Civil and Transportation Engineering, Hohai University, Nanjing, 210098 People’s Republic of China; 4 School of Civil Engineering, University of Queensland, Brisbane, 4067 Australia; 5 School of Materials Engineering (School of Environmental Engineering), Changzhou Institute of Industry Technology, Changzhou, 213164 People’s Republic of China

**Keywords:** Ecological modelling, Limnology

## Abstract

The vertical migration and accumulation of *Microcystis* colonies is a critical process in algal bloom formation. This work explored the effect of wind and light intensity on the vertical migration of *Microcystis* colonies. The wind-driven currents, light-driven changes in mass density of colonies, and the effect of colony size was coupled to simulate the vertical motion of colonies via Ansys Fluent and MATLAB. Results showed that light causes *Microcystis* to exhibit a ‘day-sinking and night-floating’ (d-n) phenomenon, however, wind weakens the phenomenon by forming a turbulent drag force that inhibits the vertical movement of *Microcystis*. This study proposed a kinetic ratio-based method, that there is a specific equilibrium turbulent kinetic energy and when turbulent kinetic energy of the water body is greater than the equilibrium turbulent kinetic energy, the d-n phenomenon does not occur. For Lake Taihu, the wind-driven turbulent kinetic energy is usually greater than the equilibrium turbulent kinetic energy. Therefore, *Microcystis* colonies may not exhibit the d-n phenomenon. Our findings provide a new theoretical basis for current process-based models in simulating algal blooms in large shallow lakes.

## Introduction

Algal blooms caused by lake eutrophication are of a global environmental and human health concern, threatening our drinking water supplies, and the ecological and economic sustainability of our freshwater ecosystems^1^. *Microcystis* is generally the dominant algal species in eutrophic lakes. Its strong vertical migration ability allows *Microcystis* to compete better than other algae for light and nutrients by occupying an optimized position^2^. Lake Taihu is the third largest freshwater lake in China, with an area of 2360 km^2^, and algal blooms have a serious impact on the lake environment. In Lake Taihu, *Microcystis* comprises more than 85% of the summer phytoplankton biomass^3^. Therefore, studying the vertical movement of *Microcystis* is essential for understanding its growth and treatment for eliminating algal blooms. Three factors typically affect the floating/sinking of *Microcystis* in water: the mass density of the *Microcystis* population^4^, wind-driven currents^5^, and *Microcystis* colony size^6^.

Ibelings et al.^7^ noticed that changes in light intensity affect photosynthesis and respiration processes of *Microcystis*, resulting in variations in the mass density of colonies. The buoyancy force varies with mass density, causing the *Microcystis* to sink gradually during the day and float at night. Previous studies cultured *Microcystis* aeruginosa^8^ and cyanobacteria purified from the field^9^ in the laboratory. Under varying light intensities, the gas vesicle expansion pressure, mass density, intracellular carbohydrate mass, and protein content were tested. Evidently, the variations in the mass density of *Microcystis* were driven by light intensity. The main reason for the variations in mass density was the variation in intracellular carbohydrate mass in *Microcystis* cells as a result of photosynthesis and respiration processes. Visser et al.^10^ determined the relationship between the rates of density change in *Microcystis* and changes in photon irradiance in laboratory experiments, and provided critical information for simulations of the ‘day-sinking and night-floating’ (d-n) phenomenon of *Microcystis* cultures in still water.

Wind-driven currents changed the pattern of the d-n phenomenon of *Microcystis* in still water^11^. George et al.^12^ proposed a concept of critical wind speed, and only when the actual wind speed is below the critical wind speed, *Microcystis* can float to the surface of the water, and sink to the deeper layers vice versa^13^. Critical wind speed was observed in different parts of the world, such as Nakdong River^14^, Lake Dianchi^15^, Mickeysloe Bay^16^, Lake Kinneret^17^, Lake Taihu^18^. The critical wind speed was different in each in-situ investigation.

The size of colonies affects the ability of *Microcystis* to resist disturbance by wind-driven currents. Medrano et al.^11^ suggested that the larger the colony size of *Microcystis*, the more resistant it is to the disturbance of wind-driven currents, which indicates that large colonies can congregate at the water surface under increased wind-speed conditions. Wu et al.^19^ observed that *Microcystis* in Lake Taihu, with a colony size of 36–120 µm, were evenly distributed vertically under windy conditions and congregated at the surface when there was no wind. Laboratory test results from Xiao et al.^20^ and a numerical simulation test from Zhao et al.^21^ showed that the ability of *Microcystis* to resist the disturbance of wind-driven currents is positively correlated with colony size. *Microcystis* has a resistance limit for turbulent kinetic energy (TKE). When TKE generated by wind is greater than the resistance limit, *Microcystis* cannot easily float on the surface.

In summary, mass density of colonies, colony size, as well as wind-driven current play critical roles in the floating–sinking process of *Microcystis*, while the mass density is partially driven by light and the turbulence was partially driven by wind. The multiple effects of wind and light essentially affect the *Microcystis* floating–sinking process. Therefore, it is important to investigate this issue. Li et al.^22^ found that turbulent mixing may effectively reduce the colony size of *Microcystis*. Chien et al.^23^ considered the effects of light-driven mass density variations and *Microcystis* colony size on the floating and sinking of *Microcystis* under hydrostatic conditions and suggested that the d-n phenomenon only occurs in large *Microcystis* colonies. Medrano et al.^24^ integrated the effects of *Microcystis* colony buoyancy variations and turbulent disturbances and proposed that *Microcystis* would be evenly distributed vertically when the effects of turbulent disturbance dominated. Liu et al.^25^ studied the effects of wind-driven currents and *Microcystis* colony size on floating velocity. The vertical distribution of *Microcystis* in Milford Lake, USA was measured and simulated, and it was proposed that the mixing process caused by wind-driven currents had an important effect on the vertical distribution of *Microcystis*. Although these studies considered the combined effects of multiple factors on *Microcystis* floating/sinking, the motions were simulated based on diffusion–dispersion models, which did not incorporate the biological effects of wind disturbance and light on the mass density variations of colonies.

The mechanism of upward movement of *Microcystis*, considering both wind and light effects, needs to be clarified. Furthermore, the conditions that allow *Microcystis* to behave like a laboratory hydrostatic culture with the d-n phenomenon are unknown. Therefore, the movement patterns of *Microcystis* in lakes (i.e., Lake Taihu, China) remain unclear. This study investigated the influence of wind and light on the floating and sinking processes of *Microcystis* and studied the vertical motion of *Microcystis* colonies. To investigate the effects of wind and light on the floating and sinking movements of *Microcystis*, we assessed the influence of the intensity of wind-driven currents and changing mass density on floating and sinking movements.

## Methods

### Model overview

The intensity of wind-driven currents was simulated using Ansys Fluent, and compared with the in situ measurements. Disturbance of wind-driven currents, light-driven variations in mass density and the effect of colony size were coupled when simulating the vertical motion of *Microcystis* using MATLAB. We used SPSS 20 to analyze correlation of the data. Details about simulations can be seen in Appendix A (Supplementary).

### In-situ investigation

Lake Taihu (30°55ʹ40ʺ–31°32ʹ58ʺ N; 119°52ʹ32ʺ–120°36ʹ10ʺ E) is located in the lower part of the Yangtze River Delta, China. It is a well-known large, shallow, and eutrophic lake. In 2017, we used an acoustic Doppler current profiler (ADCP) at two sampling sites in Meiliang Bay and Gonghu Bay in northern Lake Taihu to measure the stratified TKE in seven layers under different wind speeds throughout the year. Details about in-situ investigation and data analysis can be seen in Appendix A. Supplementary data.

### Simulations

#### Simulation of the intensity of wind-driven currents

The wind-driven currents were simulated based on the multiphase flow Eulerian model in Ansys Fluent, which allows for the modeling of multiple separate, yet interacting phases. Momentum exchange between atmosphere and water is based on the value of the fluid–fluid exchange coefficient. The volume fraction follows the continuity equation. The turbulence intensity of wind-driven currents was simulated based on the ‘realizable k–ε model’^26^. To ensure the convergence and accuracy of the algorithm, the solution method in the SIMPLEC algorithm with pressure–velocity coupling and the discrete second-order upwind format was used.

Wind stress determines the efficiency of kinetic energy transfer between the atmosphere and water, and the morphology of surface waves. The strength of the wind stress strongly depends on the average wind speed at the water surface^27^. The wind stress in the wind-driven current model is simulated as follows^28^:

1 $${F}_{x}=\frac{{\rho }_{air}}{{\rho }_{water}}{C}_{d}{u}_{wind}\sqrt{{{u}_{wind}}^{2}+{{v}_{wind}}^{2}}$$

2 $${F}_{y}=\frac{{\rho }_{air}}{{\rho }_{water}}{C}_{d}{v}_{wind}\sqrt{{{u}_{wind}}^{2}+{{v}_{wind}}^{2}}$$

where ${F}_{x}$ and ${F}_{y}$ are wind stresses; ${\rho }_{air}$ is the air density; ${\rho }_{water}$ is the water density; ${C}_{d}$ is the drag coefficient of wind stress^29^; and ${u}_{wind}$ and ${v}_{wind}$ are the components of wind speed in the horizontal plane.

Details about simulations can be seen in Appendix A. Supplementary data.

#### Simulation of the influence of wind-driven currents on the *Microcystis* floating and sinking process

Movement of the *Microcystis* colony in turbulence can be considered analogous to the sediment transport process in turbulent currents^21^. The turbulent force, ${F}_{w}$, on the *Microcystis* population under wind-driven current disturbance is composed of turbulent drag, virtual mass, and pressure-gradient forces.

The upward direction is regarded as the forward direction; thus, the formula^30^ for the turbulent force, ${F}_{w}$, per unit mass of the *Microcystis* population is represented as follows:

3 $${f}_{w}=\frac{{F}_{w}}{m}=\frac{3\mu {C}_{D}{Re}_{d}}{4{\rho }_{p}{{d}^{2}}_{p}}\left(v-u\right)+\frac{1}{2}{\left(\frac{\rho }{{\rho }_{p}}\right)}^{2}v\frac{\partial v}{\partial y}\frac{d}{dt}(v-u)$$

where $\rho$ and ${\rho }_{p}$ are the mass densities of water and *Microcystis*, respectively; $v$ and $u$ are the vertical velocities (Vy) of water and *Microcystis* colony, respectively; ${Re}_{d}$ is the Reynolds number of colonies, which is simulated as ${Re}_{d}=\frac{\rho {d}_{p}\left|v-u\right|}{\mu }$; ${C}_{D}$ is the drag coefficient of turbulent flow^30^, and ${d}_{p}$ is the *Microcystis* colony size.

To analyze the interaction between the *Microcystis* population and the continuously generated and dissipated eddies in the wind-driven current and to reflect the randomness of turbulent flow^31^, a random walk model was used to calculate the time step in this study.

Details` about simulations can be seen in Appendix A. Supplementary data.

#### Simulation of the influence of light on *Microcystis* floating and sinking

The effect of light on the floating and sinking of *Microcystis* colonies is mainly reflected in the light-driven variations in the mass density and thus in the population buoyancy. Visser et al.^10^ proposed a relationship between the variations in the mass density of *Microcystis* in still water and photon irradiance. In this study, the mass density under light-driven conditions was simulated based on the relationship between light intensity and mass density of *Microcystis*. The initial mass density^32^ of the *Microcystis* population was set to 985 kg m^−3^.

#### Simulation of the kinetic ratio for *Microcystis* floating and sinking

According to the force analysis, the effect of light can be expressed in terms of the mass density force, *F*_*ρ*_, and the effect of wind can be expressed in terms of the turbulent disturbance force, *F*_*w*_. The ratio of these two forces is defined as the kinetic ratio, *k*, which determines the dominance of wind or light in the vertical movement of *Microcystis*. Equation (4) describes *k* as follows:

4 $$k=\left|\frac{{F}_{w}}{{F}_{\rho }}\right|=\left|\frac{{f}_{w}}{{f}_{\rho }}\right|$$

where ${f}_{w}$ is the turbulent flow disturbance force per unit mass of the *Microcystis* colony (Eq. 3); ${f}_{\rho }$ is the mass density force per unit mass of the *Microcystis* colony, ${f}_{\rho }=g\frac{\left(\rho -{\rho }_{p}\right)}{{\rho }_{p}}$; and $\rho$ and ${\rho }_{p}$ are the mass densities of water and *Microcystis* colonies, respectively.

Details about simulations can be seen in Appendix A. Supplementary data.

### Parameters

#### Parameter selection

1. Wind speed
  Diurnal wind speed in Lake Taihu from January 1, 1956 to September 30, 2019 was obtained from the China Meteorological Data Network (http://www.data.cma.cn). In the past 70 years, the average diurnal wind speed in the Lake Taihu area was 3.39 m s^−1^. From the diurnal wind speed frequency distribution, the typical wind speed was 0–5 m s^−1^, accounting for 93.3% of the measurements. Therefore, wind speeds of 1, 2, 3, 4, and 5 m s^−1^ and an extremely high value of 10 m s^−1^ were selected to cover the wide range of wind speeds for the simulation of wind-driven currents.
2. Light intensity.
  In still water, the light intensity varied at different depths. According to the Lambert–Beer law, the light intensity, *I,* at a water depth, *y,* is simulated as follows^10^:
  5 $${{I}}_{{y}} ={{I}}_{{S}}/{{e}}^{{\eta y}}$$
  6 $${{I}}_{{S}}={{I}}_{{MAX}}{{\sin}}{(}{\pi t}{/}{{D}}_{{L}}{)}$$
  where η is the extinction coefficient, set at − 2 m^−1^; ${{I}}_{{MAX}}$ is the maximum light intensity at noon, set at 1000 μmol; and ${{D}}_{{L}}$ is the duration of light, set at 12 h.
3. *Microcystis* colony size
  The size and mass density of *Microcystis* colonies were determined from samples collected from Meiliang Bay and Gonghu Bay in the northern Lake Taihu. The average colony size of *Microcystis* in Lake Taihu was 342.7 µm^33^. In the study, values of 100, 300, 500, and 1000 µm were selected as four typical colony sizes of *Microcystis* in simulations, representing small to large *Microcystis* colonies. In each simulation, the size of *Microcystis* colony remains unchanged.
4. Number of *Microcystis* colony
  Considering the randomness of turbulence, the migration of 1000 colonies of the same size was simulated under the same wind speed. The colony whose final position was the median value of 1000 colonies was selected as the typical colony.

#### Computational domain

**Figure 1:**
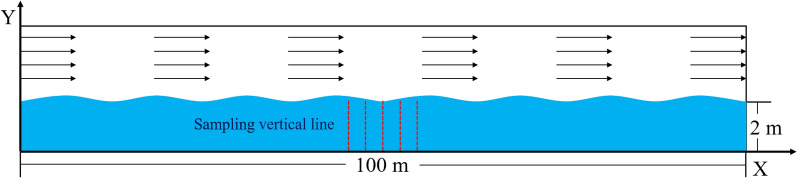
Schematic diagram of the mathematical model for wind-driven current simulations.

The two-dimensional vertical mathematical model in Ansys Fluent for the simulation of different strengths of wind-driven currents is shown in Fig. 1. A water depth of 2 m was used to emulate the actual water depth of Lake Taihu. The bottom height was set to 0 m. To guarantee the complete simulation of wind-driven currents, the fetch was set to 100 m. The strength of the wind field on the water surface was determined by the simulated wind speed that remained unchanged during the simulation. To weaken the influence of offshore flow on the intensity of the wind-driven current, we considered the average value recorded on five vertical lines as the final strength of the wind-driven currents. Vertical lines at 5-m intervals were established in the middle of the water body, and data were recorded (X = 40 m, 45 m, 50 m, 55 m, and 60 m). The average value was used to evaluate the effect of wind-driven currents on the floating–sinking of *Microcystis*.

In addition to wind speed, wind-driven currents are generally affected by fetch and duration^27^. To achieve practical results, the duration of the simulation was set to 48 h, and why? Due to the variance of wind-driven currents during the early stage, the simulated results of wind-driven currents in the 24–48 h period were used. The simulation time in following sections was counted from the 24 h. The effects of wind and light on the floating and sinking of *Microcystis* were considered in the model. To analyze the multiple effects of wind and light on the floating and sinking process of *Microcystis*, the vertical movement of different *Microcystis* colonies was simulated for 24 h under different intensities of wind-driven current disturbances and light intensity variations. The wind-driven current data for the 0–24 h period are represented as data from the 24 to 48 h period in the following sections. The light intensity was set to be the same as described in the section above, and the *Microcystis* colonies were initially placed on the water surface.

## Results

### Results of wind-driven current simulation

**Figure 2:**
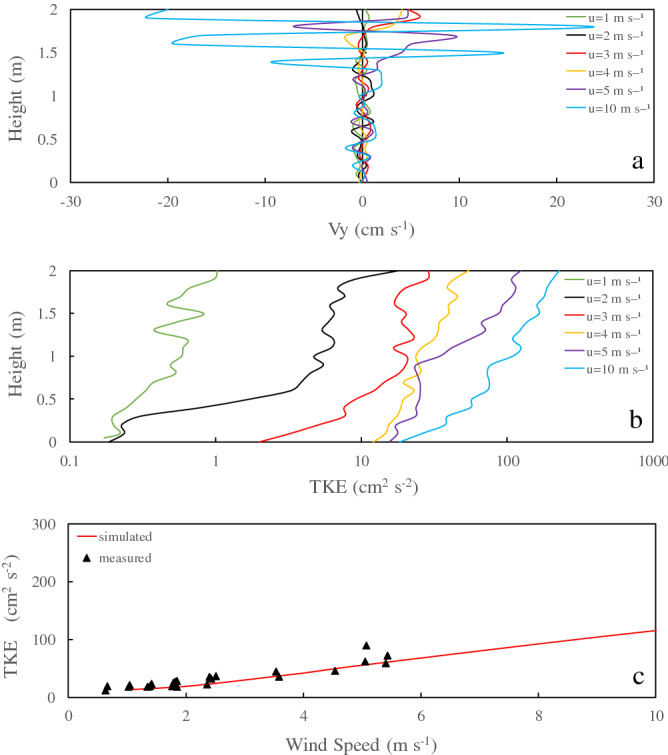
Simulated structure of wind-driven currents (**a**), relationship between depth and TKE (**b**), relationship between TKE and wind speed (**c**).

The distribution of velocity in the Y direction (Vy, Fig. 1) and of Turbulent kinetic energy (TKE) at 12 h from the start of the selected simulation and the comparison between the simulation and measured data at Lake Taihu (Fig. 2). The Vy of wind-driven currents oscillated within a certain frequency and had a conspicuous peak value (Fig. 2a). With an increase in wind speed, the oscillation ranges of Vy of wind-driven currents also increased. The magnitude of the wind-generated vertical flow velocity gradually decreases with an increase in water depth. Within 50 cm below the water surface, the oscillation range of the wind-generated vertical flow velocity was substantial, which changed rapidly. At depths between 50 and 200 cm below the water surface, the oscillation ranges of the wind-generated vertical flow velocity decreased. As the wind speed increased, the degree of turbulence in wind-driven currents increased simultaneously (Fig. 2b, Table 1). The wind-driven TKE gradually decreased with increasing water depth.

**Table 1:** Velocity and TKE of wind-driven currents at different depths.

Wind speed (m s^−1^)	Value					
	y = 2 m		y = 1 m		y = 0.5 m	
	Vy (cm s^−1^)	TKE (cm^−2^ s^−2^)	Vy (cm s^−1^)	TKE (cm^−2^ s^−2^)	Vy (cm s^−1^)	TKE (cm^−2^ s^−2^)
1	0.26	1.03	− 0.18	0.58	0.07	0.28
2	− 0.30	18.04	− 0.15	4.74	− 0.10	1.72
3	3.03	28.82	0.27	20.55	0.11	9.16
4	3.88	54.41	0.29	23.59	0.15	19.49
5	4.03	123.74	0.45	34.43	0.64	25.06
10	− 16.14	227.05	− 0.62	108.89	1.26	57.34

The average values measured from different layers in the water column were compared with the simulated mean TKEs of the entire water column of the wind-driven current at 12 h (Fig. 2c). The average TKE of the measured wind-driven currents in the entire water column was correlated with wind speed. The simulated results and measured data show a similar pattern.

### Effect of wind on *Microcystis* floating and sinking

**Figure 3:**
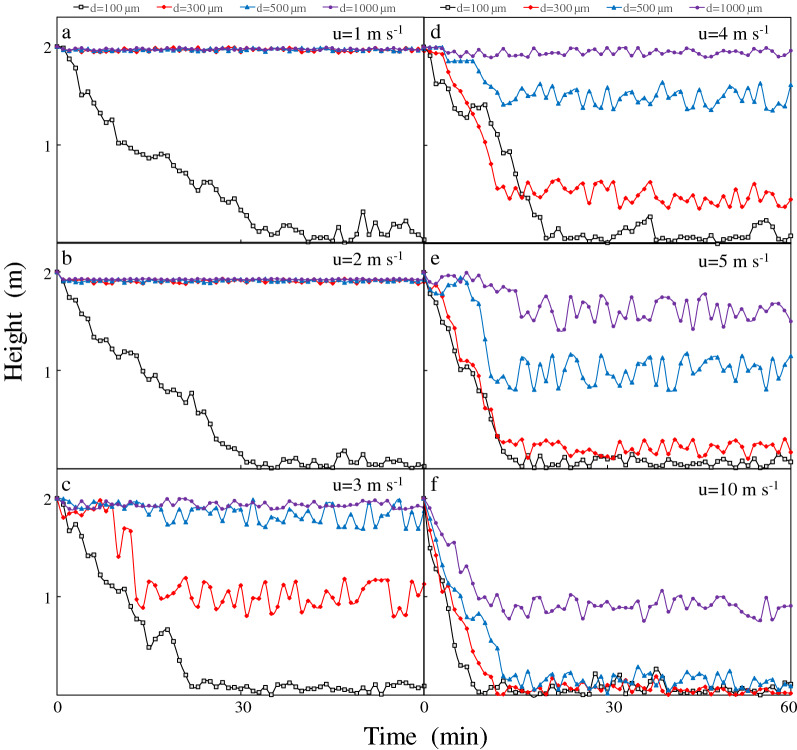
Vertical migration of *Microcystis* colonies at various constant wind speeds: (**a**) 1 m s^−1^, (**b**) 2 m s^−1^, (**c**) 3 m s^−1^, (**d**) 4 m s^−1^, (**e**) 5 m s^−1^, and (**f**) 10 m s^−1^.

Disregarding the effect of light, the mass density of colony was set to a constant value of 985 kg m^−3^^32^, and the effect of wind on the *Microcystis* floating and sinking process was simulated. Firstly, different sizes of colonies were placed on the water surface, and the vertical migration was simulated under the different intensities of wind-driven currents. Table 2 shows the median value and standard deviation of the final position of *Microcystis* colonies in the water column at the end of simulation. We found that with the increase of wind speed, the median value of the final position with the same size decreased after 1 h (0–1 h), and the final position with the smaller particle size was closer to the bottom. Besides, with the increase of wind speed, the standard deviation of the final position increases after 1 h, indicating a greater measure of dispersion. The migration trajectory of the typical colony was plotted in Fig. 3.

**Table 2:** Median value and standard deviation of the final position of *Microcystis* colonies under various constant wind speeds.

Wind speed	Diameter			
	100 µm	300 µm	500 µm	1000 µm
1 m s^−1^	0.83/0.67	1.90/0.42	1.94/0.22	1.96/0.13
2 m s^−1^	0.72/0.70	1.82/0.51	1.93/0.32	1.93/0.18
3 m s^−1^	0.55/0.74	1.30/0.60	1.67/0.36	1.88/0.23
4 m s^−1^	0.46/0.81	1.01/0.64	1.50/0.46	1.87/0.39
5 m s^−1^	0.37/0.86	0.75/0.69	1.01/0.52	1.62/0.42
10 m s^−1^	0.20/0.99	0.33/0.88	0.47/0.70	1.06/0.64

Median value/standard deviation (unit: m).

The vertical variation patterns that were simulated by placing *Microcystis* colonies of different sizes at the water surface under different intensities of wind-driven current disturbance (Fig. 3). The wind speed remained unchanged under different simulation conditions. Two *Microcystis* movement patterns, Mode I and Mode II, were observed. Mode I resisted the disturbance of wind-driven currents allowing *Microcystis* to continue floating on the water surface, whereas Mode II could not resist the disturbance of wind currents; *Microcystis* sank and was turbulent in the water column. When the wind speed was extremely high (u = 10 m s^−1^), the colonies of all tested sizes were in Mode II. When the wind speed was the lowest (u = 1 m s^−1^), only the small colonies (d = 100 µm) sank (Mode II), and the remaining colonies were in Mode I. As the wind speed increased, the movement of smaller colonies changed from Mode I to Mode II, and they sank; larger colonies maintained their resistance to the wind-driven current disturbance.

**Figure 4:**
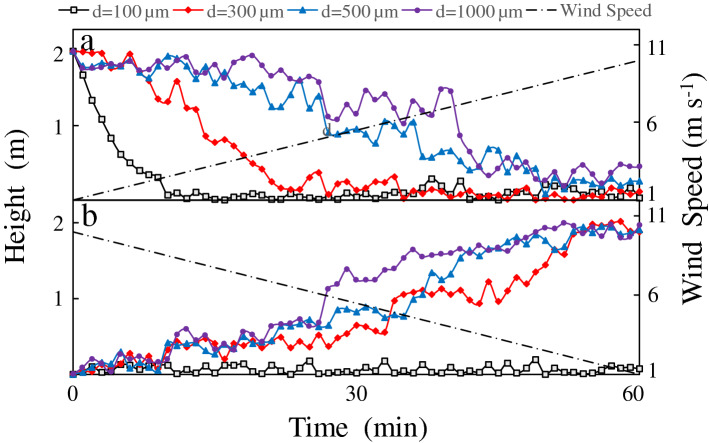
Vertical migration of *Microcystis* colonies under varying wind speeds: (**a**) simulations in which colonies were place on the water surface and (**b**) simulations in which colonies were placed at the base of the water column.

The effect of variations in the intensity of wind field on the floating and sinking of *Microcystis* was not well understood. The behaviors of two groups of *Microcystis* were simulated using varying wind field strengths. In the first group, the *Microcystis* colonies were placed on the water surface, and the wind speed was increased from 1 to 10 m s^−1^ for simulating *Microcystis* sinking. In the second group, *Microcystis* colonies were placed at the water bottom, and the wind speed was decreased from 10 to 1 m s^−1^ to simulate *Microcystis* floating. Table 3 shows the median value and standard deviation of the final position of *Microcystis* colonies in the water column at the end of simulation. We found that with the increase of wind speed, the final position of colonies with the same particle size after 1 h was consistent with that under constant wind speed. The migration trajectory of the typical colony was plotted in Fig. 4.

**Table 3:** Median value and standard deviation of the final position of *Microcystis* colonies under varying wind speeds.

Wind speed	Diameter			
	100 µm	300 µm	500 µm	1000 µm
1–10 m s^−1^	0.23/0.95	0.35/0.81	0.51/0.62	1.11/0.55
10–1 m s^−1^	0.82/0.68	1.91/0.46	1.92/0.28	1.94/0.15

Median value/standard deviation (unit: m).

**Figure 5:**
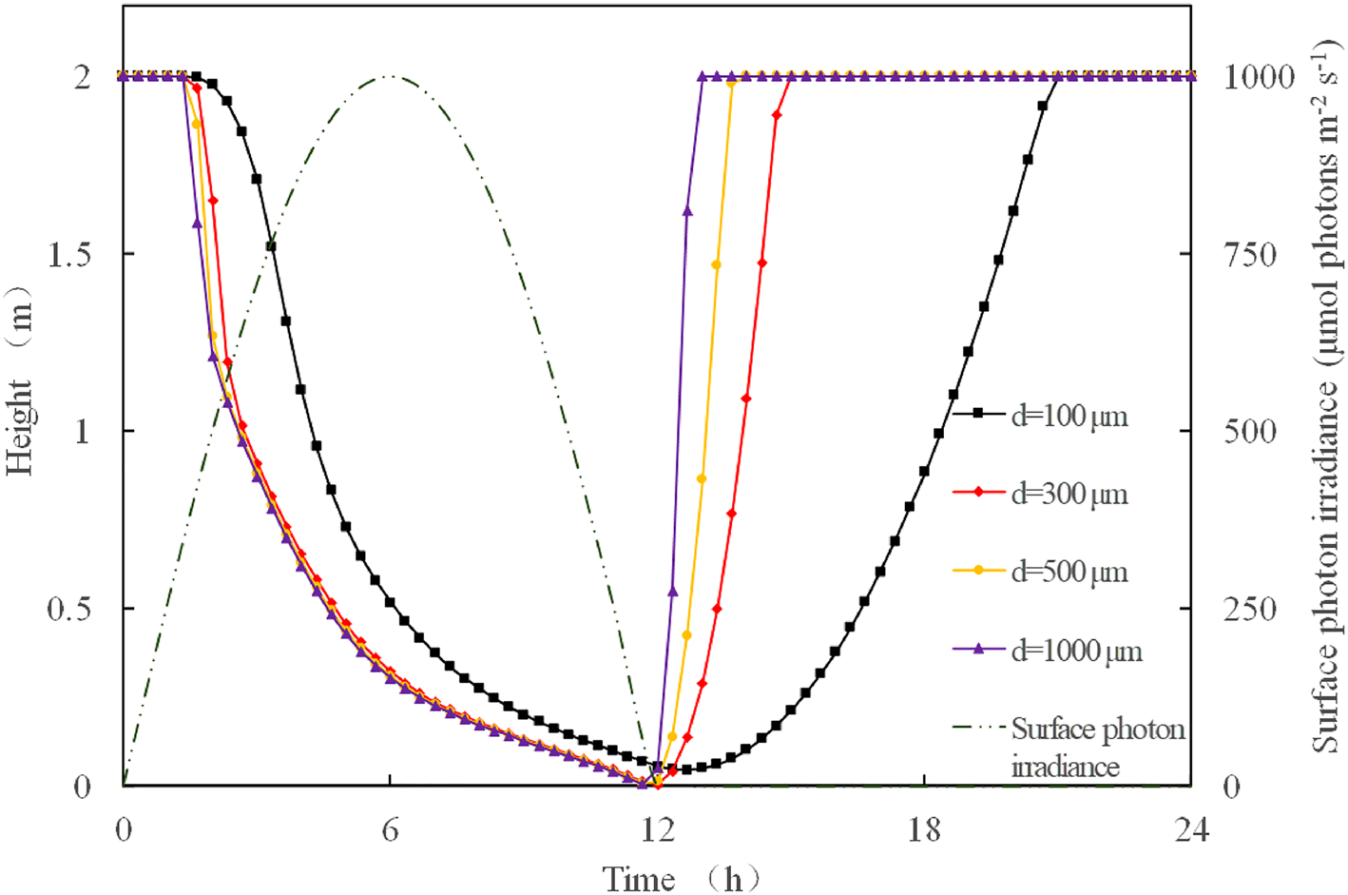
Vertical migration of *Microcystis* colonies under varying light intensity.

Results indicate that the movement patterns of the *Microcystis* colonies under varying wind intensities were the same as those with constant wind (Fig. 4). Small colonies (d = 100 µm) maintained Mode II movements during the simulation. Even when the colonies were initially on the water surface (Fig. 5a), they exhibited sinking in the short term. Medium-sized colonies (d = 300 µm) could float on the water surface with Mode I movement, when the wind speed was low (u < 3 m s^−1^). When the wind speed was higher than 3 m s^−1^, the colonies could not resist the wind-driven current and sank into the water with Mode II movement. Colonies with large sizes (d = 500 and 1000 µm) were more resistant to wind-driven current disturbances and remained on the surface longer than in the other scenarios. When the wind speed was low, they came up to the water surface and exhibited Mode I movement. However, under extremely high wind speeds, the colonies sank into the water and exhibited Mode II movement.

### Effect of light on *Microcystis* floating and sinking

The effect of variation in light intensity on the mass density of *Microcystis* colonies was included in the model. The light intensity was set according to the variation in light intensity within a day by setting 0–12 h as the time with light and 12–24 h as the time without light. *Microcystis* colonies were placed on the water surface at the beginning of the day, and the vertical movement of the *Microcystis* colonies with different colony sizes was simulated with the change of light intensity over 24 h.

The result shows that the variations in light intensity can affect the vertical movement of *Microcystis* (Fig. 5). In the first 12 h of light exposure, all *Microcystis* colonies started to sink after approximately 2 h of exposure, eventually reaching the bottom. In the first 12 h without light, all *Microcystis* colonies floated to the surface due to reduced mass density. All the *Microcystis* colonies showed the ‘day-sinking and night-floating’ (d-n) phenomenon when simulating with the variations in day and night light intensities. The floating and sinking speeds of large *Microcystis* colonies were faster than those of smaller colony sizes. When simulating the floating and sinking speeds of *Microcystis* colonies in still water using Stokes’ formula, the mass density driven by the variation of light intensity played a key role^8^.

### Multiple effect of wind and light on the *Microcystis* floating and sinking process

**Figure 6:**
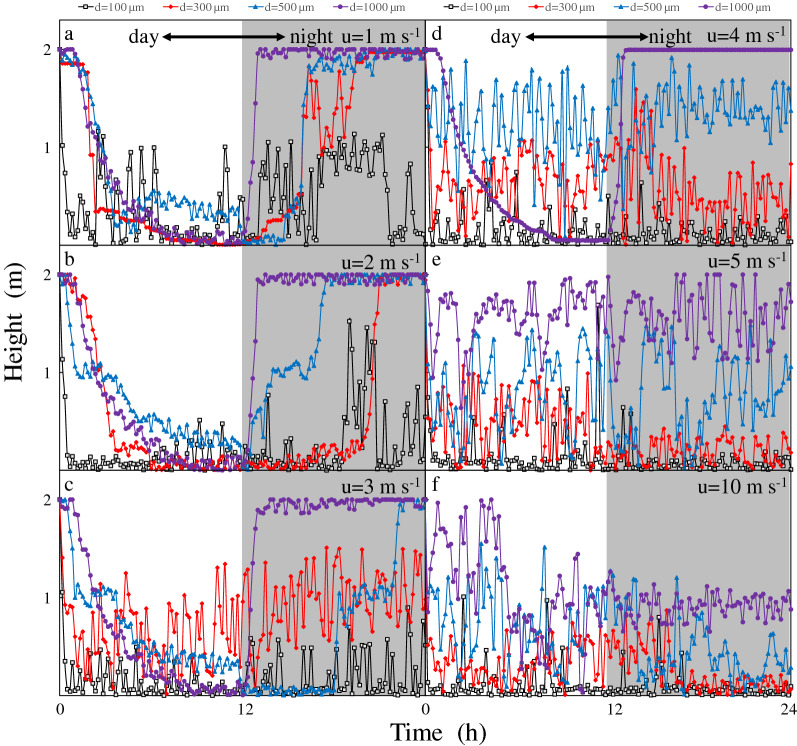
Vertical migration of *Microcystis* colonies under the effect of wind and light: (**a**) 1 m s^−1^, (**b**) 2 m s^−1^, (**c**) 3 m s^−1^, (**d**) 4 m s^−1^, (**e**) 5 m s^−1^, and (**f**) 10 m s^−1^.

Table 4 shows the median value and standard deviation of the final position of *Microcystis* colonies in the water column at the end of simulation. We found that with the increase of wind speed, the final position of colonies with the same particle size after 24 h was consistent with the law above. The migration trajectory of the typical colony was plotted in Fig. 6.

**Table 4:** Median value and standard deviation of the final position of *Microcystis* colonies under various constant wind speeds in 24 h.

Wind speed	Diameter			
	100 µm	300 µm	500 µm	1000 µm
1 m s^−1^	0.85/0.72	1.89/0.44	1.90/0.23	1.93/0.15
2 m s^−1^	0.73/0.75	1.79/0.50	1.89/0.30	1.92/0.21
3 m s^−1^	0.55/0.79	1.32/0.61	1.65/0.38	1.87/0.24
4 m s^−1^	0.48/0.80	1.02/0.66	1.46/0.47	1.85/0.34
5 m s^−1^	0.40/0.90	0.73/0.71	1.05/0.50	1.67/0.45
10 m s^−1^	0.23/1.01	0.38/0.91	0.42/0.73	1.09/0.68

Median value/standard deviation (unit: m).

The result shows that the smaller colonies with d = 100 μm did not show the ‘day-sinking and night-floating’ (d-n) phenomenon under the several given wind speed conditions, and the influence of wind was dominant (Fig. 6). When the wind speed was less than 3 m s^−1^, the medium-sized colonies (d = 300 μm) exhibited the d-n phenomenon, which is similar to the behavior under hydrostatic conditions. When the wind speed was higher than 3 m s^−1^, the phenomenon no longer occurred. The d-n phenomenon was still observed in colonies with large sizes (d = 500 μm and 1000 μm), when the wind speed was higher than 3 m s^−1^. However, when the wind speed was greater than 4 m s^−1^, no colonies showed the phenomenon. From the diurnal migration trajectory of *Microcystis*, as the wind speed increased, the position of *Microcystis* in the water column gradually approached the bottom.

## Discussion

**Figure 7:**
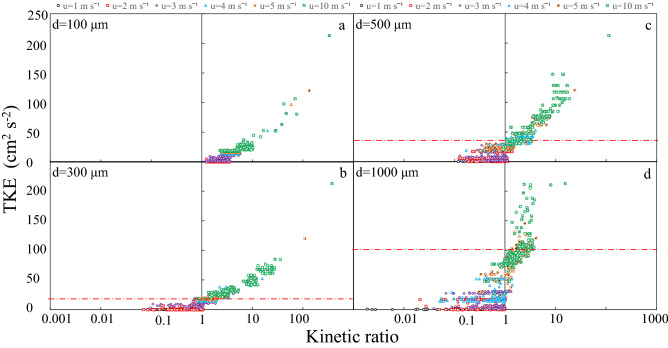
Relationship between kinetic ratio and TKE: (**a**) 100 µm, (**b**) 300 µm, (**c**) 500 µm, (**d**) 1000 µm.

According to Eq. (4), when the kinetic ratio (*k*) > 1, the effect of wind dominates, and when *k* < 1, the effect of light becomes dominant. When the effect of wind dominated, the turbulent drag force dominated the vertical movement, and the more colonies were trapped in the water layer. When the effect of light dominated, the change in mass density drove the sinking and floating process of the colony, which led to the tendency of the ‘day-sinking and night-floating’ (d-n) phenomenon. Light had a greater effect on the floating and sinking of larger colonies while in comparison, smaller colonies were more affected by wind disturbance. Under the effects of wind and light, the average kinetic ratio of *Microcystis* with different colony sizes was shifted by wind-driven currents. By calculating the average kinetic ratio, we analyzed the dominant factors of *Microcystis*’ vertical movement, which was affected by intensities of light and wind. The variations of light intensity were observed to affect the mass density of *Microcystis* colonies. During the day, photosynthesis caused increasing mass density, and *Microcystis* colonies tend to sink. The average TKE at the corresponding location is shown in Fig. 7. Under the constant effect of wind-driven currents, the kinetic ratios of different colony sizes varied considerably. When the colony size was < 100 μm, the kinetic ratio was generally < 1, indicating that the wind played a dominant role. When the colony size reached 1000 μm or more, the effect of light could still play a dominant role, unless when the wind speed was greater than 5 m s^−1^. For each colony size, an equilibrium point was observed in which the effects of light and wind negated each other; a wind speed less than this point indicated light dominance, and vice versa, which is defined as ‘equilibrium TKE’.

*Microcystis* colonies tend to float at night when the mass density decreases. The vertical velocity of wind-driven currents oscillates within a certain frequency and has a conspicuous peak value, the oscillation ranges of the vertical velocity of wind-driven currents also increases with the increase in wind speed. Hence, the water turbulence produces a drag force on the colonies, which acts in opposition to the direction of their movement. This means that wind-driven currents create an upward drag force when *Microcystis* sinks during the day, and a downward drag force is created when colonies rise at night. The magnitude of the drag force is related to the strength of the turbulence, and the ability of colonies to resist the drag force is highly dependent on the square of the colony size (Eq. 3), which also indicates that the smaller colonies are more dispersed in the water column under the same wind speed.

**Figure 8:**
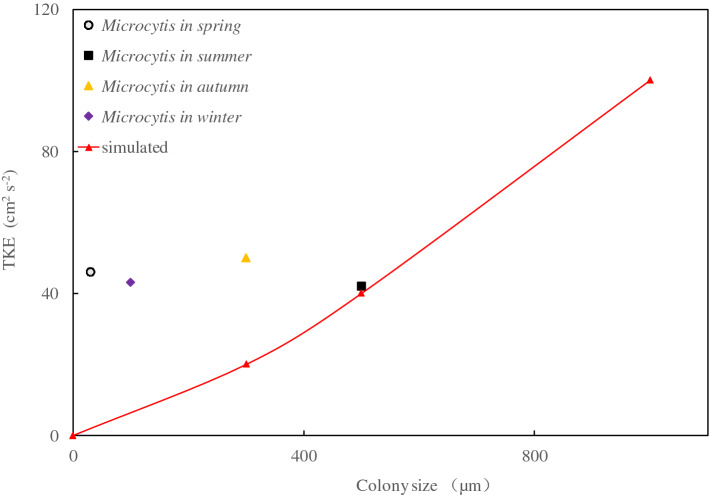
Relationship between colony sizes and equilibrium TKE.

Realistically, a lake cannot be absolutely still. *Microcystis* populations generally begin to grow in spring and become smaller after autumn. The illumination time and light intensity above the water surface variations depend on the season and weather in Lake Taihu^34^. The TKE corresponding to normal colony sizes and wind speeds in Lake Taihu throughout the year are plotted in Fig. 8 and listed in Table 2. The relationship between *Microcystis* colony size and equilibrium TKE obtained from this study is shown as a solid line. Most *Microcystis* colonies can grow to 300–400 µm in April^33^. According to the actual wind speed statistics, the average wind speed in April was 3.6 m s^−1^, and the measured TKE strength was approximately 45 cm^2^ s^-2^, which was greater than the equilibrium TKE. Therefore, wind dominated the vertical movement of *Microcystis*. There was less time for the d-n phenomenon, and *Microcystis* was distributed in the water layer. From July to August, the colony size was approximately 500 µm^35^, the average wind speed was 3.4 m s^−1^, and the measured TKE was approximately 42 cm^2^ s^−2^. Although it was closer to equilibrium TKE, the wind-dominated period was longer. The daytime measured results^36^ showed that large colonies float easily on the water surface, whereas small colonies are mixed in the water layer. With wind speeds of 2–3 m s^−1^, this effect does not change owing to the light conditions. Although wind has a dominant effect most of the time, light can play a dominant role in the floating and sinking of *Microcystis* during relatively still periods. However, when the wind speed increases, the drag force of turbulent currents starts to dominate. Thus, it is difficult for light to alter the trajectory of *Microcystis*.

The method proposed in this study, which combines the influence of wind-driven currents and the change in mass density of *Microcystis* on the migration of *Microcystis*, has broad applicability in the field of algal bloom prediction in shallow lakes in the future. For other shallow lakes, such as Lake Chaohu^37^ and Lake Dianchi^15^, the influence of wind-driven currents on the formation of water blooms has received more attention. This study provides an effective simulation method and provides theoretical guidance for this field. However, in deep lakes, such as Lake Erie^38^ and Lake Xiapu^39^, turbulence caused by temperature stratification cannot be ignored. Besides, the formation of algal blooms decreases the transparency of the water column and influences the vertical distribution of light intensity, which suggests a positive feedback regulation of *Microcystis* surface scum formation and stability by self-shading^40^. In our follow-up study, this mechanism will be further explored.

## Conclusions

In conclusion, *Microcystis* tends to exhibit the ‘day-sinking and night-floating’ (d-n) phenomenon from the changing light intensities, however, wind currents generate turbulent drag forces that prevent the vertical movement of *Microcystis* and weaken this d-n phenomenon. Colonies with smaller sizes are less resistant to turbulence and are more dispersed in the water column. The existence of the d-n phenomenon can be determined comparing the kinetic ratio and the equilibrium TKE. When the TKE of the water body is greater than the equilibrium TKE, the d-n phenomenon does not occur for *Microcystis*. For Lake Taihu, the *Microcystis* colonies do not exhibit the d-n phenomenon, because the effect of wind dominates the vertical movement of *Microcystis*. In the absence of this phenomenon, the *Microcystis* colonies remain in the still water layer where the TKE is less than their equilibrium TKE. Our method highlights the use of a critical threshold of the kinetic ratio, which help simplify the numerical simulation and forecasting of *Microcystis* blooms.

## Supplementary Information

Supplementary Information.

## Data Availability

The datasets used and/or analyzed during the current study are available from the corresponding author upon reasonable request. Equipment and settings: All figures were created in Excel 2016.
