# Supplementary material for: Influence of wind and light on the floating and sinking process of Microcystis
Source: Sci Rep. 2022 Apr 5;12:5655. doi: 10.1038/s41598-022-08977-5 (PMC8983747; doi:10.1038/s41598-022-08977-5)
Supplement: Supplementary file 1 — Supplementary Information. [file 41598_2022_8977_MOESM1_ESM.docx]

**Influence of Wind and Light on the Floating and Sinking Process of *Microcystis***

**Appendix A. Supplementary data**

**1. Calculation of wind-driven currents**

The Eulerian multiphase model in ANSYS Fluent allows for the modeling of multiple separate, yet interacting phases. The phases can be liquids, gases, or solids in nearly any combination. An Eulerian treatment is used for each phase, in contrast to the Eulerian-Lagrangian treatment that is used for the discrete phase model.

The description of multiphase flow as interpenetrating continua incorporates the concept of phasic volume fractions, denoted here by $\alpha_{q}$. Volume fractions represent the space occupied by each phase, and the laws of conservation of mass and momentum are satisfied by each phase individually. The derivation of the conservation equations can be done by ensemble averaging the local instantaneous balance for each of the phases. The volume of phase q, $V_{q}$, is defined by:

$V_{q}=\int\alpha_{q}dV$ （S1）

where $\sum_{q=1}^{n} \alpha_{q}=1$.

The equation of conservation of mass is:

$\frac{\partial}{\partial t}\left( \alpha_{q}\rho_{q} \right)+\nabla\left( \alpha_{q}\rho_{q}v_{q} \right)=\sum_{p=1}^{n} (m_{pq}-m_{qp})$ (S2)

where $\alpha_{q}$ is the volume fraction of each phase, $\rho_{q}$ is the mass density of each item,$v_{q}$ is the velocity of phase, $m_{pq}$ is the mass transferred from p phase to q phase.

The equation of conservation of momentum is:

$\frac{\partial}{\partial t}\left( \alpha_{q}\rho_{q}v_{q} \right)+\nabla\left( \alpha_{q}\rho_{q}{v_{q}}^{2} \right)=-\alpha_{q}\nabla p+\nabla\tau_{q}+\alpha_{q}\rho_{q}g+\sum_{p=1}^{n} \left( F_{pq}+m_{pq}v_{pq}-m_{qp}v_{qp} \right)+F_{wq}+F_{tq}$ (S3)

where $\tau_{q}$ is the surface tension of the liquid phase, g is the acceleration of gravity,$F_{pq}$ is the drag force of p phase to q phase, which is defined as wind stress in this calculation. $F_{wq}$ is the wall lubrication force with the boundary, $F_{tq}$ is the turbulence force.

The realizable $k-\varepsilon$ model differs from the standard $k-\varepsilon$ model in two important ways:

(1) The realizable - model contains an alternative formulation for the turbulent viscosity.

(2) A modified transport equation for the dissipation rate,$\varepsilon$, has been derived from an exact equation for the transport of the mean-square vorticity fluctuation.

The modeled transport equations for k and $\varepsilon$ in the realizable $k-\varepsilon$ model are:

$\frac{\partial}{\partial t}\left( \rho k \right)+\frac{\partial}{\partial x_{j}}\left( \rho ku_{j} \right)=\frac{\partial}{\partial x_{j}}\left[ \left( \mu+\frac{\mu_{t}}{\sigma_{k}} \right)\frac{\partial k}{\partial x_{j}} \right]+G_{k}+G_{b}-\rho\varepsilon-Y_{M}+S_{k}$ (S4)

$\frac{\partial}{\partial t}\left( \rho\varepsilon\right)+\frac{\partial}{\partial x_{j}}\left( \rho\varepsilon u_{j} \right)=\frac{\partial}{\partial x_{j}}\left[ \left( \mu+\frac{\mu_{t}}{\sigma_{\varepsilon}} \right)\frac{\partial\varepsilon}{\partial x_{j}} \right]+\rho C_{1}S\varepsilon-\rho C_{2}\frac{\varepsilon^{2}}{k+\sqrt{v\varepsilon}}+C_{1\varepsilon\frac{\varepsilon}{k}}C_{3\varepsilon}G_{b}+S_{\varepsilon}$ （S5）

$C_{1}=max\left[ 0.43,\frac{\eta}{\eta+5} \right]$ (S6)

$\eta=S\frac{k}{\varepsilon}$ (S7)

$S=\sqrt{2S_{ij}S_{ij}}$ (S8)

where $G_{k}$ is the turbulent kinetic energy generated by the average velocity gradient, $G_{b}$ is the turbulent kinetic energy generated by buoyancy, $Y_{M}$ is the contribution of undulating expansion to the total dissipation rate in compressible turbulence, $\sigma_{k}$ and $\sigma_{\varepsilon}$ are turbulent Prandtl numbers, $\sigma_{k}$ is 1.0 and $\sigma_{\varepsilon}$ is 1.2, $\mu_{t}$ is vortex viscosity, $S_{k}$ and $S_{\varepsilon}$ are user-defined source terms.

**2. Iterative calculations using MATLAB**

All equations in this section are calculated using MATLAB R2018, and the upward direction is regarded as the forward direction.

The position of *the Microcystis* population in the water column (y) is described as follows:

$\text{y}\text{ }\text{=}\text{ }\text{y}_{\text{0}}\text{+}\text{S}$ (S9)

where $\text{y}_{\text{0}}$ is the initial position of the *Microcystis* population in the water column, and $\text{S}$ is the total displacement.

$\text{S}\text{ }\text{=}\text{Δ}\text{S}_{\text{1}}\text{+}\text{Δ}\text{S}_{\text{2}}\text{+}\text{⋯}\text{+}\text{Δ}\text{S}_{\text{n}}$ (S10)

$\text{Δ}\text{S}_{\text{i}}\text{ }\text{=}\text{ }u_{\text{i}}\text{×Δ}\text{t}$ (S11)

where $u_{\text{i}}$ is the velocity of the *Microcystis* population, and $\text{Δ}\text{t}$ is the time step controlled by the random walk model, as shown in Equation S12.

$\text{Δ}\text{t}\text{=}\min\left\{ \left( \tau=-T_{L}log(r) \right)，\left( t_{cross}=-\tau In\left[ 1-\left( \frac{L_{e}}{\tau\left| \text{u}_{\text{i}}-v \right|} \right) \right] \right) \right\}$ （S12）

where r is a random number uniformly distributed in the interval (0,1), $T_{L}=0.15\frac{k}{\varepsilon}$, $L_{e}$ is the length of the vortices, and $\text{u}_{\text{i}}$ and $v$ are the vertical velocity of water and *Microcystis* colony, respectively.

$\text{u}_{\text{i}}=\text{u}_{\text{i-1}}+a_{\text{i}}\text{×Δ}\text{t}$ （S13）

where $a_{\text{i}}$ is the acceleration of the *Microcystis* population, and $u_{\text{0}}$ is the initial velocity of the *Microcystis* population, which is 0 m s^-1^.

$a_{\text{i}}=\frac{\text{F}_{\text{h}}}{m}$ （S14）

where $\text{F}_{\text{h}}$ is the resultant force of the *Microcystis* population, and $m$ is the mass of the *Microcystis* population.

$\text{F}_{\text{h}}=F_{w}+F_{f}$（S15）

where $F_{w}$ is the turbulent force of the *Microcystis* population, which is calculated using Equation 3 in the manuscript; $F_{f}$ is the buoyancy force of the *Microcystis* population.

**3. Calculation of the kinetic ratio *k***

All equations in this section are calculated using MATLAB R2018, and the upward direction is regarded as the forward direction.

The kinetic ratio *k* is calculated according to Equation 4 in the manuscript:

$k=\left| \frac{F_{w}}{F_{\rho}} \right|=\left| \frac{f_{w}}{f_{\rho}} \right|$ （4）

where $f_{w}$ is the turbulent force per unit mass of the *Microcystis* colony (Equation 3), $f_{\rho}$ is the mass density force per unit mass of the *Microcystis* colony, and $\rho$ and $\rho_{p}$ are the mass densities of the water and *Microcystis* colonies, respectively.

$f_{w}=\frac{F_{w}}{m}$ （S16）

where $F_{w}$ is the turbulent force of the *Microcystis* population, which is calculated using Equation 3 in the manuscript;$m$ is the mass of the *Microcystis* population.

$f_{\rho}=\frac{F_{f}}{m}=g\frac{\left( \rho-\rho_{p} \right)}{\rho_{p}}$ （S17）

where $F_{f}$ is the buoyancy force of the *Microcystis* population, $\rho$ and $\rho_{p}$ are the mass densities of the water and *Microcystis* colonies, respectively.

**4. In-situ investigation**

4.1 Field Site

Lake Taihu (30°55ʹ40ʺ-31°32ʹ58ʺ N; 119°52ʹ32ʺ-120°36ʹ10ʺ E) is located in the lower part of the Yangtze River Delta, China. Our study was performed in Meiliang Bay and Gonghu Bay within the north portion of Lake Taihu. Meiliang Bay has a surface area of 119.71 km^2^. Gonghu Bay has a surface area of 169.83 km^2^. The average water depth of both bays is 1.9 m. In 2017, we used an acoustic Doppler current profiler (ADCP) at two sampling sites in Gonghu Bay (sampling site 1, 31°24ʹ13ʺN; 120°20ʹ6ʺE) and Meiliang Bay (sampling site 2, 31°28ʹ57ʺN; 120°10ʹ52ʺE) in northern Lake Taihu to measure the stratified TKE in seven layers under different wind speeds once a month.

4.2 Collection of wind fields

The data of wind speed and direction was collected from the Wuxi weather station (31°27ʹ19ʺN; 120°15ʹ33ʺE) at intervals of 1 h. Since the time we used ADCP to measure the stratified TKE at each sampling site was fixed to 1 h, we assumed that the wind speed and direction remained unchanged during the sampling. Table S1 shows the data of wind speed and direction during the sampling.

Table S1 Wind speed and direction

| Month | Sampling Site | | | |
| --- | --- | --- | --- | --- |
|  | sampling site 1 | | sampling site 2 | |
|  | Wind speed  (m s^-1^) | Wind direction  (°) | Wind speed  (m s^-1^) | Wind direction  (°) |
| 1 | 1.03 | 288 | 1.05 | 253 |
| 2 | 0.63 | 179 | 0.67 | 100 |
| 3 | 5.05 | 203 | 5.07 | 174 |
| 4 | 5.40 | 100 | 5.43 | 57 |
| 5 | 3.59 | 150 | 4.54 | 177 |
| 6 | 2.43 | 201 | 2.40 | 155 |
| 7 | 3.54 | 300 | 2.51 | 288 |
| 8 | 1.85 | 7 | 2.36 | 105 |
| 9 | 1.77 | 50 | 1.42 | 121 |
| 10 | 1.85 | 103 | 1.82 | 217 |
| 11 | 1.80 | 183 | 1.79 | 117 |
| 12 | 1.35 | 70 | 1.39 | 37 |

4.3 Measurements of wind-driven currents

During sampling, the ADCP (FlowQuest 2000, San Diego, USA) was fixed on the motor boat, and the position of sampling site was determined by a handheld GPS. The ADCP configuration was set as 0.13 m in bin cells. The vertical velocity and direction in seven water layers were monitored in 0.13 m intervals from the surface to the bottom of the lake, with a standard deviation of 1 mm/s. Due to the unreceived reflection of 0.27 m near the ADCP and the height of the ADCP, the blind zone from the surface to a depth of 1.10 m could not be monitored in this study. The movement of the motor boat was automatically corrected by the ADCP while the vertical velocity of the wind-driven current was recorded. The total sampling time is 1 h, with a standard interval of 2 s.

The stratified TKE in each water layer was calculated by the equation S18, and the average TKE of wind-driven currents in the whole water column was calculated by the equation S19. Figure S1 shows the monthly variations of the average TKE.

${TKE}_{i}=\frac{1}{2}\left( v^{'} \right)^{2}$ (S18)

Where ${TKE}_{i}$ is the stratified TKE in each water layer, $v^{'}$ is the fluctuating velocity recorded by the ADCP.

${TKE}_{average}=\frac{\sum_{1}^{7} {TKE}_{i}}{7}$ (S19)

where ${TKE}_{average}$ is the average TKE of wind-driven currents in the whole water column, ${TKE}_{i}$ is the stratified TKE in each water layer calculated by the equation S18.

Figure S1 Monthly variations of the average TKE （a~l represent Jan to Dec）
